# Age attenuates the T‐type Ca_V_3.2‐RyR axis in vascular smooth muscle

**DOI:** 10.1111/acel.13134

**Published:** 2020-03-18

**Authors:** Gang Fan, Mario Kaßmann, Yingqiu Cui, Claudia Matthaeus, Séverine Kunz, Cheng Zhong, Shuai Zhu, Yu Xie, Dmitry Tsvetkov, Oliver Daumke, Yu Huang, Maik Gollasch

**Affiliations:** ^1^ Experimental and Clinical Research Center (ECRC) a joint cooperation between the Charité Medical Faculty and the Max Delbrück Center for Molecular Medicine (MDC) Charité – Universitätsmedizin Berlin Berlin Germany; ^2^ Hunan Cancer Hospital The Affiliated Cancer Hospital of Xiangya School of Medicine Central South University Changsha China; ^3^ Crystallography Max‐Delbrück‐Center for Molecular Medicine Berlin Germany; ^4^ Electron Microscopy Facility Max Delbrück Center for Molecular Medicine (MDC) Berlin Germany; ^5^ Institute of Chemistry and Biochemistry Freie Universität Berlin Berlin Germany; ^6^ Institute of Vascular Medicine and School of Biomedical Sciences Chinese University of Hong Kong Hong Kong China; ^7^ Medical Clinic for Nephrology and Internal Intensive Care Charité – Universitätsmedizin Berlin Berlin Germany; ^8^ Department of Geriatrics University Medicine Greifswald Greifswald Germany

**Keywords:** aging, calcium sparks, caveolae, ryanodine receptors, T‐type calcium channels, vascular smooth muscle

## Abstract

Caveolae position Ca_V_3.2 (T‐type Ca^2+^ channel encoded by the α‐3.2 subunit) sufficiently close to RyR (ryanodine receptors) for extracellular Ca^2+^ influx to trigger Ca^2+^ sparks and large‐conductance Ca^2+^‐activated K^+^ channel feedback in vascular smooth muscle. We hypothesize that this mechanism of Ca^2+^ spark generation is affected by age. Using smooth muscle cells (VSMCs) from mouse mesenteric arteries, we found that both Ca_v_3.2 channel inhibition by Ni^2+^ (50 µM) and caveolae disruption by methyl‐ß‐cyclodextrin or genetic abolition of Eps15 homology domain‐containing protein (EHD2) inhibited Ca^2+^ sparks in cells from young (4 months) but not old (12 months) mice. In accordance, expression of Ca_v_3.2 channel was higher in mesenteric arteries from young than old mice. Similar effects were observed for caveolae density. Using SMAKO Ca_v_1.2^−/−^ mice, caffeine (RyR activator) and thapsigargin (Ca^2+^ transport ATPase inhibitor), we found that sufficient SR Ca^2+^ load is a prerequisite for the Ca_V_3.2‐RyR axis to generate Ca^2+^ sparks. We identified a fraction of Ca^2+^ sparks in aged VSMCs, which is sensitive to the TRP channel blocker Gd^3+^ (100 µM), but insensitive to Ca_V_1.2 and Ca_V_3.2 channel blockade. Our data demonstrate that the VSMC Ca_V_3.2‐RyR axis is down‐regulated by aging. This defective Ca_V_3.2‐RyR coupling is counterbalanced by a Gd^3+^ sensitive Ca^2+^ pathway providing compensatory Ca^2+^ influx for triggering Ca^2+^ sparks in aged VSMCs.

## Graphical abstract

Cav3.2‐RyR axis in smooth muscle cell is down‐regulated by age‐related ultrastructural alterations of caveolae and reduced Cav3.2 expression. This defective Cav3.2‐RyR coupling is counterbalanced by a Gd3+ sensitive Ca2+ pathway providing compensatory Ca2+ influx for triggering Ca2+ sparks in aged VSMCs.

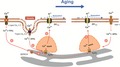

## 1 INTRODUCTION

In resistance arteries, voltage‐dependent Ca^2+^ channels activate ryanodine receptors (RyRs) to cause elementary Ca^2+^ release events (Ca^2+^ sparks) from the sarcoplasmic reticulum (SR) (Essin et al., 2007; Jaggar et al., 1998; Nelson et al., 1995; Wang et al., 2004). Ca^2+^ release from the SR in the form of Ca^2+^ sparks opens numerous large‐conductance Ca^2+^‐sensitive K^+^ (BK_Ca_) channels, causing spontaneous transient outward K^+^ currents (STOCs) (Knot, Standen, & Nelson, 1998; Nelson et al., 1995). As a result, Ca^2+^ spark–BK_Ca_ channel coupling induces vascular smooth muscle cell (VSMCs) hyperpolarization and attenuation of arterial constriction (Brenner et al., 2000; Löhn et al., 2001; Pérez, Bonev, Patlak, & Nelson, 1999). In previous studies, we demonstrated that L‐type Ca_V_1.2 channels play the predominant role (~75%) in Ca^2+^ sparks generation in mesenteric arterial VSMCs, and T‐type Ca_V_3.2 channels, localized in caveolae, represent an additional source (~25%) (Fan, Kaßmann, Hashad, Welsh, & Gollasch, 2018; Hashad et al., 2018). In the latter pathway, caveolae position Ca_V_3.2 channels sufficiently close to RyRs (<40 nm) of the sarcoplasmic reticulum (SR) for extracellular Ca^2+^ influx to trigger Ca^2+^ sparks and large‐conductance Ca^2+^‐activated K^+^ channel feedback in vascular smooth muscle (Fan et al., 2018; Harraz et al., 2014; Hashad et al., 2018; Löhn et al., 2000). These conclusions were mainly derived from experiments using Ca_V_3.2 channel (*Cacna1h* ^−/−^) and caveolin‐1 (*Cav1* ^−/−^) knockout mice. Although genetic caveolin‐1 deletion leads to a complete lack of caveolae from the VSMC plasma membrane, data interpretation is limited because *Cav1* deletion may affect SR Ca^2+^ load and is known to increase the density of BK_Ca_ channels in VSMCs (Cheng & Jaggar, 2006). Caveolins affect also trafficking of other K^+^ channels (K_v_1.5) to cholesterol‐rich membrane microdomains (McEwen, Li, Jackson, Jenkins, & Martens, 2008).

Little is known about the effects of aging on the T‐type Ca_V_3.2‐RyR axis to generate Ca^2+^ sparks. While L‐type Ca^2+^ current densities are preserved in VSMCs, aging has been reported to cause decrements in Ca^2+^ signaling in response to either ryanodine receptor stimulation by caffeine or inositol trisphosphate (InsP_3_) receptor activation with phenylephrine in mesenteric arteries of mice (del Corsso et al., 2006). Loss of Ca_V_3.2 channels attenuates a protective function to excess myogenic tone in response to intravasal pressure (Mikkelsen, Björling, & Jensen, 2016). Advanced age can also alter the composition of lipid rafts and caveolae, which could affect a variety of signaling molecules (Bergdahl & Sward, 2004; Parton & Simons, 2007) to contribute to the pathophysiology of Alzheimer's, Parkinson's, diabetes, and cardiovascular diseases (Boersma et al., 2001; Headrick et al., 2003; Ohno‐Iwashita, Shimada, Hayashi, & Inomata, 2010; Simons & Ehehalt, 2002). Aging has been also found to alter the number and morphology of caveolae in smooth muscle cells (Bakircioglu et al., 2001; Lowalekar, Cristofaro, Radisavljevic, Yalla, & Sullivan, 2012; Ratajczak et al., 2003). We hypothesize that aging affects the T‐type Ca_V_3.2‐RyR axis to generate Ca^2+^ sparks in vascular smooth muscle. To test this hypothesis, we used methyl‐ß‐cyclodextrin, smooth muscle‐specific (SMAKO) Ca_V_1.2^−/−^ mice and a novel Eps15 homology domain‐containing protein (*EHD2*) knockout mouse model, which leads to destabilization of caveolae at the plasma membrane (Lian, Matthaeus, Kassmann, Daumke, & Gollasch, 2019). We also evaluated the role of luminal SR calcium on T‐type Ca_V_3.2‐RyR coupling. Clarification of this hypothesis is important for understanding age‐dependent effects in cardiovascular disease and may provide new therapeutic avenues in the elderly.

## 2 RESULTS

### 2.1 Age effects on T‐type Ca_V_3.2‐RyR axis

**Figure 1:**
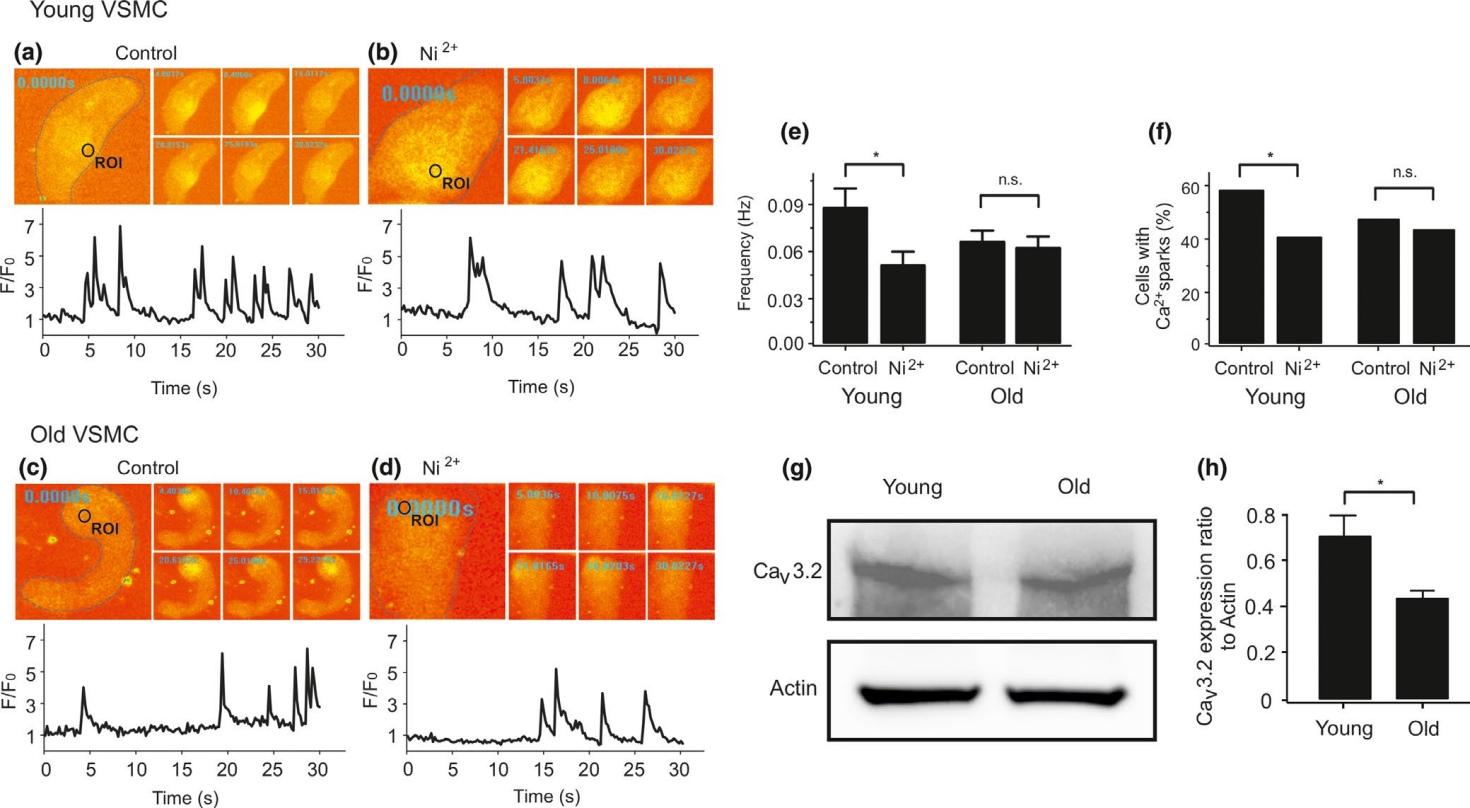
Age attenuates the role of Ca_V_3.2 channels in Ca^2+^ spark generation and decreases Ca_V_3.2 protein expression in VSMC. (a), Ca^2+^ fluorescence images of a Fluo‐4‐AM–loaded VSMC from a young mouse and time course of Ca^2+^ fluorescence changes in the cellular ROI (upper panel). Cell boundary is marked with dashed line. (b), same as (a) but in the presence of Ni^2+^ (50 µM). (c), same as (a) but in a VSMC from an old mouse. (d), same as (c) but in the presence of Ni^2+^ (50 µM). (e, f), summary of the results. Ca^2+^ spark frequency (e) and fraction of cells producing Ca^2+^ sparks (f) in VSMCs from young mice (*n* = 102), in VSMCs from young mice cells incubated with Ni^2+^ (*n* = 85), in VSMCs from aged mice (*n* = 129), and in VSMCs from aged mice cells incubated with Ni^2+^ (*n* = 127). Cells were isolated from 4 mice in each group; 25–40 cells were recorded and analyzed from each mouse. VSMC, vascular smooth muscle cell. (g), Western blot analysis of Ca_V_3.2 proteins in mesenteric arteries of young versus old mice. (h), quantification of Western blot results. Mesenteric arteries were taken from 9 mice in each group. *, *p* < .05. n.s., not significant

The T‐type Ca_v_3.2 channel blocker Ni^2+^ decreased Ca^2+^ spark frequency and fraction of cells with sparks in young VSMCs (see also (Fan et al., 2018; Hashad et al., 2018)), while it failed to decrease Ca^2+^ spark events in old VSMCs (Figure 1). These data suggest that Ca_v_3.2 channels contribute to generation of Ca^2+^ sparks in young but not in old VSMC. To address whether the reduced function of T‐type Ca_v_3.2 channels in generating Ca^2+^ sparks in old VSMCs could rely on reduced protein expression, we analyzed Ca_v_3.2 protein expression in mesenteric arteries from young mice versus old mice. In Western blot analyses, we found that Ca_v_3.2 expression decreased with age (Figure 1g,h).

### 2.2 Role of luminal SR calcium on T‐type Ca_V_3.2‐RyR axis

**Figure 2:**
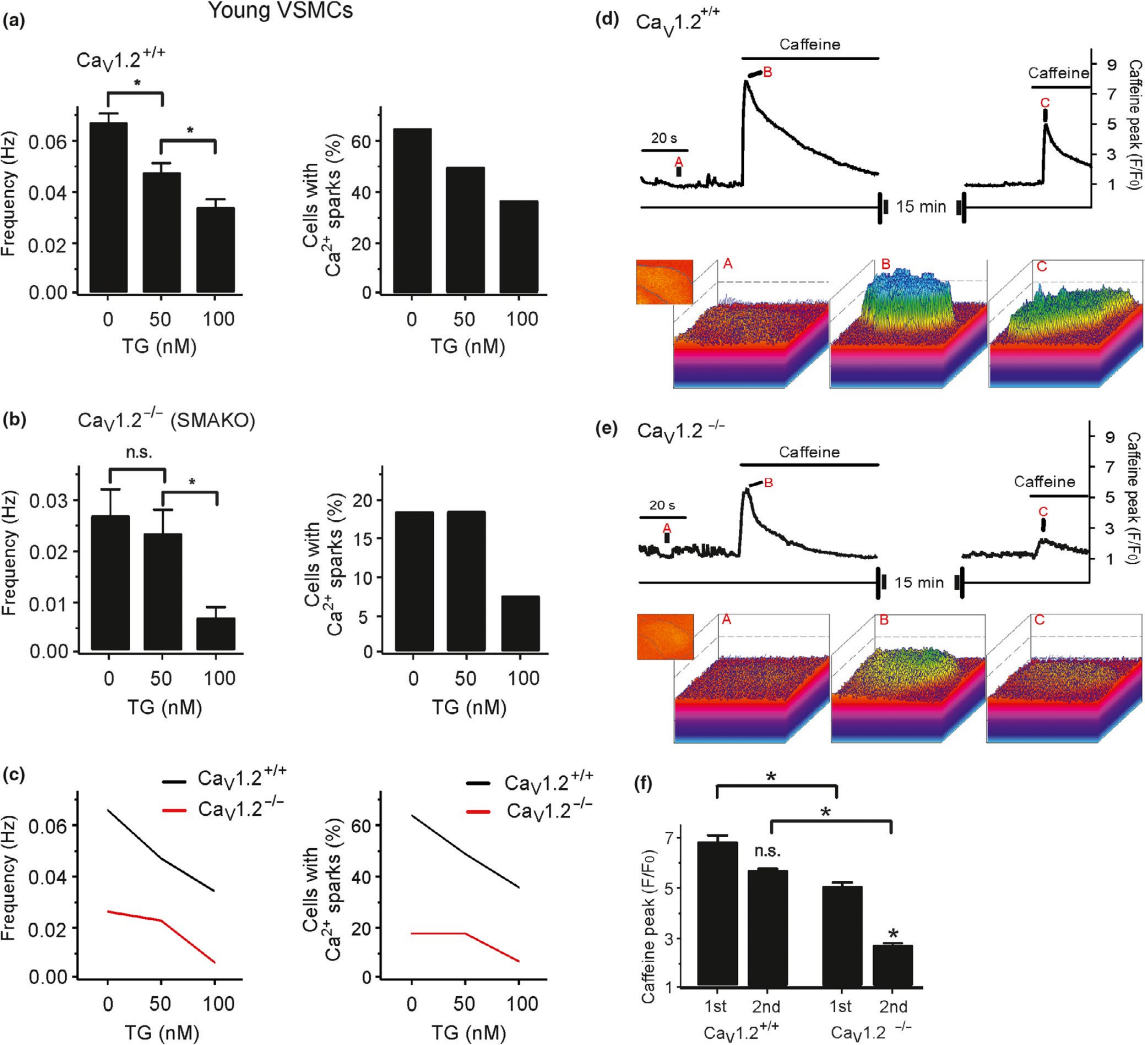
Role of luminal SR calcium on T‐type Ca_V_3.2‐RyR axis. Effects of different concentrations of thapsigargin on Ca^2+^ spark frequency (a, left) and fraction of cells producing Ca^2+^ sparks (a, right) in Ca_v_1.2^+/+^ VSMCs from young mice. Effects of different concentrations of thapsigargin on Ca^2+^ spark frequency (b, left) and fraction of cells producing Ca^2+^ sparks (b, right) in VSMCs from Ca_v_1.2^−/−^ (SMAKO) mice. (c), overlay of the data for Ca^2+^ spark frequency (left) and fraction of cells producing Ca^2+^ sparks (right). Cells were isolated from 4 mice in each group; 30–35 cells were recorded and analyzed from each mouse. (d), time course of Ca^2+^ fluorescence changes in the cellular ROI in a wild‐type (Ca_V_1.2^+/+^) Fluo‐4‐AM–loaded VSMC induced by 10 mM caffeine (upper panel) and Ca^2+^ fluorescence plots (lower panel). (e), the same as (d), but in Ca_V_1.2^−/−^ VSMC. (f), summary of the 10 mM caffeine‐induced Ca^2+^ peaks in wild‐type versus Ca_V_1.2^−/−^ VSMCs. *n* = 7 cells from 3 mice, 2–3 cells were recorded and analyzed from each mouse. *, *p* < .05. n.s., not significant

Thapsigargin inhibits the SR Ca^2+^ transport ATPase (SERCA) and thereby reduces SR [Ca^2+^] load (Janczewski & Lakatta, 1993; Sagara & Inesi, 1991; Thastrup, 1990). We studied the effects of thapsigargin on [Ca^2+^]_SR_ load and its role on T‐type Ca_V_3.2‐RyR axis. Caffeine (10 mM)‐induced peak fluorescence was measured to monitor maximal RyR Ca^2+^ release from SR stores. Our data showed that thapsigargin decreased concentration‐dependently caffeine‐induced cytosolic [Ca^2+^] peaks (Figure S1a–d). The results confirm that thapsigargin causes luminal SR calcium depletion. We next studied the individual contributions of Ca_v_1.2 versus Ca_v_3.2 channels to generate Ca^2+^ sparks under these different [Ca^2+^]_SR_ loads. We found that [Ca^2+^]_SR_ depletion by thapsigargin reduced Ca^2+^ spark frequency and the percentage of cells firing Ca^2+^ sparks in Ca_v_1.2^+/+^ VSMCs (Figure 2a,c) (see also (Essin et al., 2007)). In contrast, thapsigargin had no or little effects on Ca^2+^ spark frequency and the percentage of cells firing Ca^2+^ sparks in Ca_v_1.2^−/−^ (SMAKO) VSMCs (Figure 2b,c). These data are consistent with the idea that L‐type Ca_v_1.2 channels couple indirectly to RyRs, that is, by influencing luminal SR calcium load to generate Ca^2+^ sparks (Essin et al., 2007). The data also show that SR Ca^2+^ load is controlled by SERCA (Nelson et al., 1995). We next studied how Ca_v_1.2 channel ablation and reduced [Ca^2+^]_SR_ load affect the Ca_V_3.2‐RyR axis, that is, direct coupling between Ca_V_3.2 channels and RyRs to generate Ca^2+^ sparks (Fan et al., 2018; Hashad et al., 2018; Löhn et al., 2000). Consistent with our previous results (Essin et al., 2007), we found that [Ca^2+^]_SR_ was lower in Ca_v_1.2^−/−^ (SMAKO) VSMCs compared to Ca_v_1.2^+/+^ control cells. As illustrated in Figure 2d–f, caffeine‐induced cytosolic [Ca^2+^] peaks were larger in Ca_v_1.2^+/+^ cells compared to SMAKO Ca_v_1.2^−/−^ VSMCs, consistent with the idea that L‐type Ca_V_1.2 channels are critical for SR Ca^2+^ load and peak [Ca^2+^] release. We compared the role of Ca^2+^ uptake into SR in these cells. 15 min after the first caffeine pulse, subsequent application of caffeine induced a strong [Ca^2+^] peak in Ca_v_1.2^+/+^ control compared to Ca_v_1.2^−/−^ (SMAKO) cells (Figure 2d–f). We also compared the effects of caffeine on mesenteric arteries in the absence and presence of Ni^2+^. Ni^2+^ did not alter caffeine‐induced constrictions (Figure S1i–k). These data indicate that SR Ca^2+^ load mainly depends on Ca^2+^ influx through L‐type Ca_V_1.2 channels (see also (Essin et al., 2007)). We confirmed these results by measuring BK_Ca_ channel currents activated by Ca^2+^ sparks (STOCs) in VSMCs (Figure S1e–h). STOCs were measured in presence of Cd^2+^ and/or Ni^2+^ after depletion of the [Ca^2+^]_SR_ by thapsigargin. The holding potential was set to −40 mV, a physiological membrane potential that should drive T‐type Ca^2+^ channel‐mediated Ca^2+^ sparks, enabling the activation of BK_Ca_ channels (Fan et al., 2018; Harraz et al., 2014; Hashad et al., 2018). Figure S1 shows that thapsigargin removed ~60% of STOCs in VSMCs (Figure S1e–g). The Ca_v_1.2 channel blocker Cd^2+^ blocked all STOCs in thapsigargin‐treated cells (Figure S1f), while Ni^2+^ had no effects (Figure S1e,h). Together, the results indicate that (a) Ca^2+^ influx through L‐type Ca_V_1.2 channels is the main source of filling the SR with Ca^2+^ and (b) proper function of the T‐type Ca_V_3.2‐RyR axis requires sufficient high [Ca^2+^]_SR_ load.

### 2.3 Aging and alterations of VSMC caveolae

**Figure 3:**
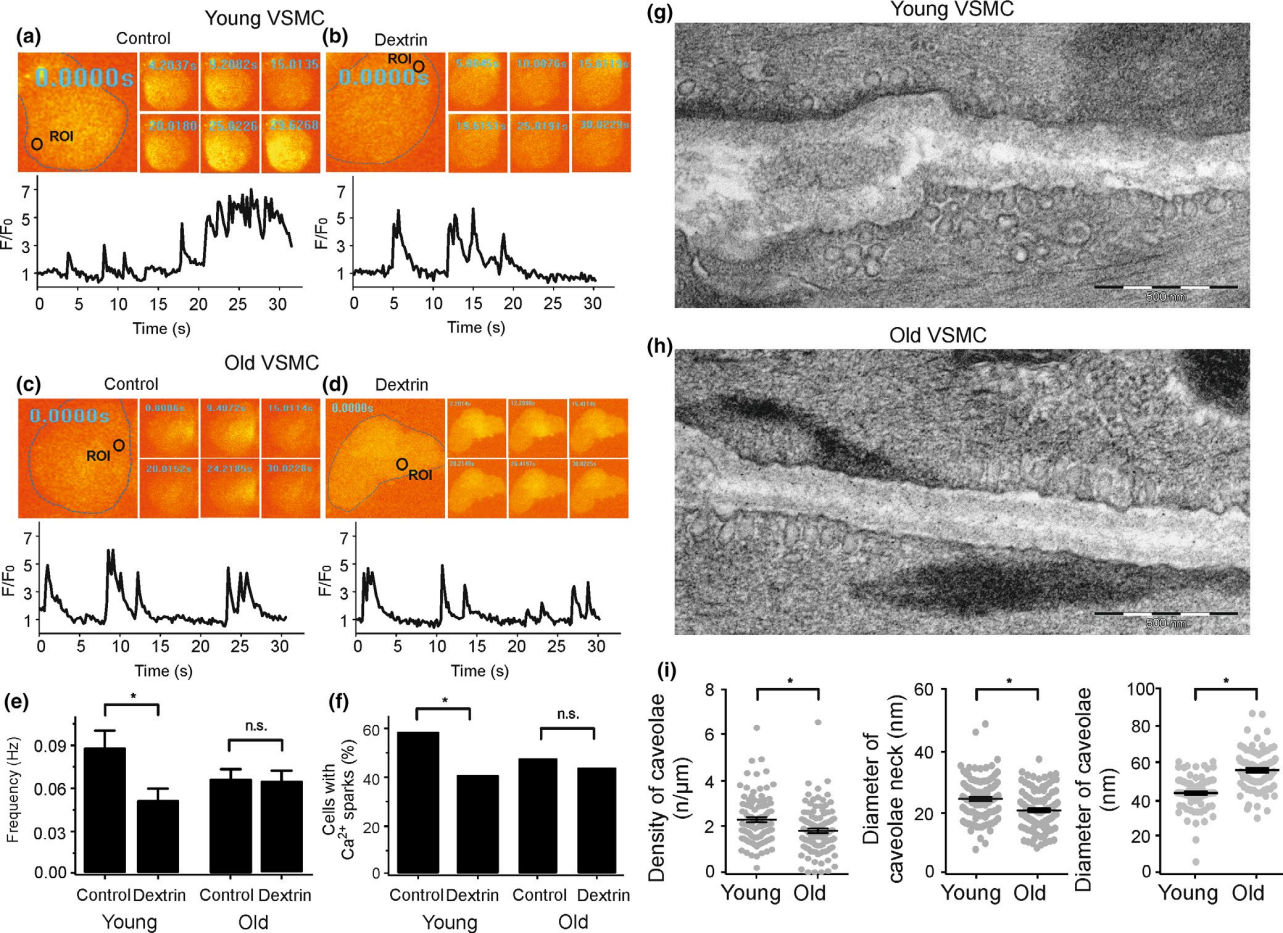
Defective Ca_V_3.2‐RyR axis in aged VSMC result from alterations in the ultrastructure of caveolae. (a), Ca^2+^ fluorescence images of a Fluo‐4‐AM–loaded VSMC from a young mouse and time course of Ca^2+^ fluorescence changes in the cellular ROI (upper panel). Cell boundary is marked with dashed line. (b), same as (a) but with a cell incubated with methyl‐ß‐cyclodextrin (10 mM, 90 min at room temperature) to disrupt caveolae. (c), same as (a) but with VSMCs from old mice. (d), same as (c) but with a cell incubated with methyl‐ß‐cyclodextrin. (e, f), summary of the results. Ca^2+^ spark frequency (e) and fraction of cells producing Ca^2+^ sparks (f) in VSMCs from young mice (*n* = 98), in VSMCs from young mice cells incubated with methyl‐ß‐cyclodextrin (*n* = 111), in VSMCs from old mice (*n* = 121), and in VSMCs from old mice cells incubated with methyl‐ß‐cyclodextrin (*n* = 128). Cells were isolated from 4 mice in each group; 25–40 cells were recorded and analyzed from each mouse. (g), Electron microscopy image of a young VSMC. (h), same as (g) but from old VSMC. (i), summary of the results. Caveolae density, diameter of caveolae neck, caveolae size in VSMCs from young versus old mice (10–20 cells from each mouse, 4 mice in each group). *, *p* < .05. n.s., not significant

**Figure 4:**
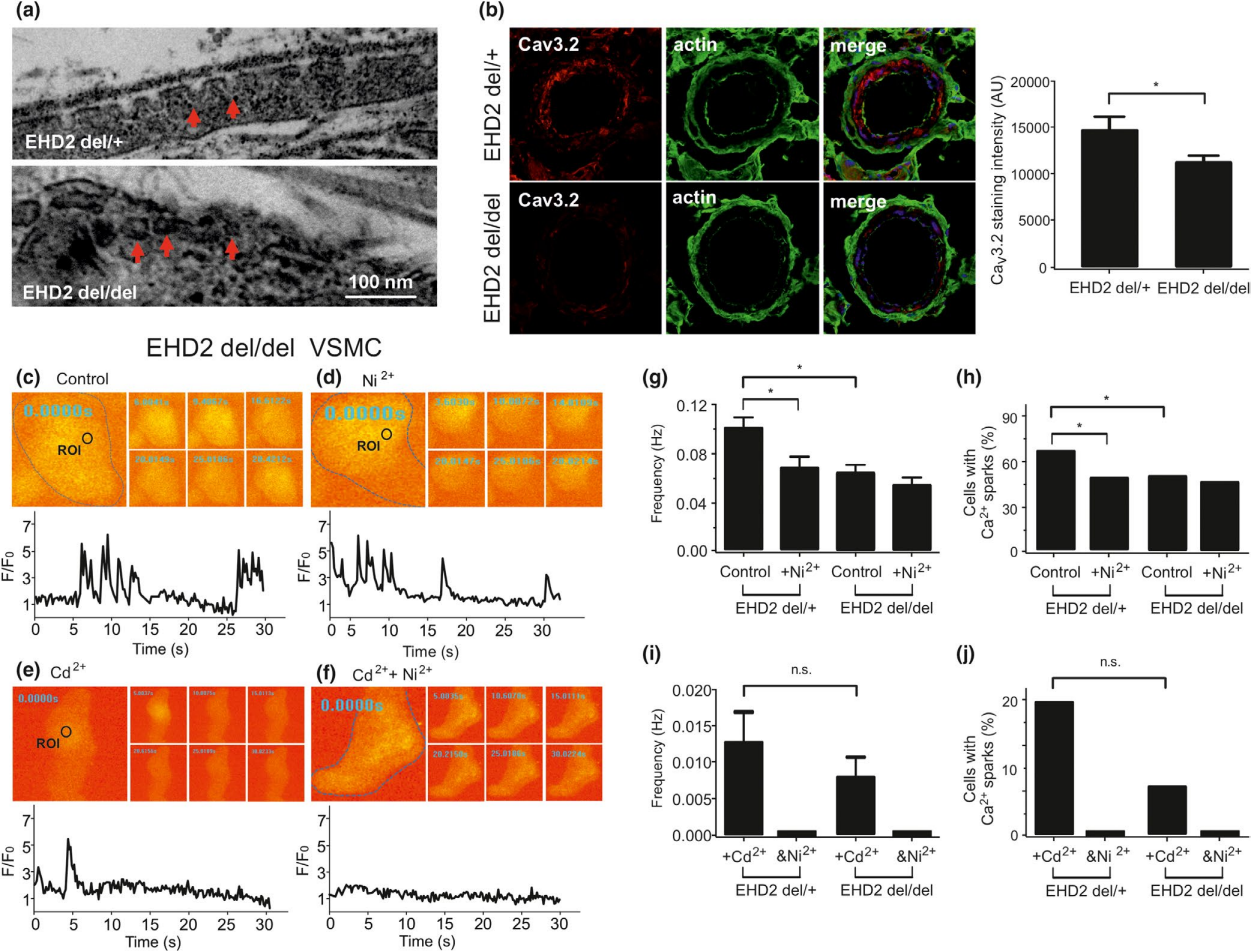
*EHD2* knockout (*EHD2* del/del) alters the ultrastructure of caveolae and decrease Ca_V_3.2 expression, resulting in Ca_V_3.2‐RyR axis malfunction. (a), Electron microscopy image of a *EHD2* del/+ VSMC and a *EHD2* del/del VSMC. (b, left), Ca_V_3.2 immuno‐staining in BAT cryostat sections from EHD2 del/+ and del/del mice. (b, right), summary of the results, *n* (del/+)=46/5 mice and *n* (del/del)=53/5 mice. (c), Ca^2+^ fluorescence images of a Fluo‐4‐AM–loaded VSMC from *EHD2* del/del mouse and time course of Ca^2+^ fluorescence changes in the cellular ROI (upper panel). Cell boundary is marked with dashed line. (d), same as (c) but in the presence of Ni^2+^ (50 µM). (e), same as (c) but in the presence of Cd^2+^ (200 µM). (f), same as (e) but in the presence of Ni^2+^ (50 µM). (g, h), summary of the results. Ca^2+^ spark frequency (g) and fraction of cells producing Ca^2+^ sparks (h) in VSMCs from *EHD2* del/+ mice (*n* = 99), in VSMCs from EHD2 del/+ mice cells incubated with Ni^2+^ (*n* = 96), in VSMCs from *EHD2* del/del mice (*n* = 144), and in VSMCs from *EHD2* del/del mice cells incubated with Ni^2+^ (*n* = 125). Cells were isolated from 4 mice in each group; 25–40 cells were recorded and analyzed from each mouse. (I, j), summary of the results. Ca^2+^ spark frequency (i) and fraction of cells producing Ca^2+^ sparks (j) in VSMCs from *EHD2* del/+ mice incubated with Cd^2+^ (*n* = 56), in VSMCs from *EHD2* del/+ mice cells incubated with Ni^2+^+Cd^2+^ (*n* = 56), in VSMCs from *EHD2* del/del mice incubated with Cd^2+^ (*n* = 75), and in VSMCs from *EHD2* del/del mice cells incubated with Ni^2+^+Cd^2+^ (*n* = 68). Cells were isolated from 4 mice in each group; 15–20 cells were recorded and analyzed from each mouse. *, *p* < .05. n.s., not significant

Defective Ca_V_3.2‐RyR axis in old VSMCs could result from alterations in the ultrastructure of caveolae, where Ca_v_3.2 channels reside to drive RyR‐mediated Ca^2+^ sparks (Fan et al., 2018; Harraz et al., 2014; Hashad et al., 2018). We first explored the contribution of caveolae to Ca^2+^ spark generation in VSMCs using methyl‐ß‐cyclodextrin (10 mM), a cholesterol‐depleting drug, which is known to disturb caveolae and inhibit a significant fraction of Ca^2+^ sparks in VSMCs (Löhn et al., 2000). In accordance with our previous data (Fan et al., 2018; Löhn et al., 2000), we found that methyl‐ß‐cyclodextrin decreased the frequency of Ca^2+^ spark and the fraction of cells with sparks by ~30% in young VSMCs. However, methyl‐ß‐cyclodextrin did not alter Ca^2+^ spark generation in old VSMCs (Figure 3a–f). Ni^2+^ (50 µM) did not further reduce Ca^2+^ sparks in methyl‐β‐cyclodextrin treated VSMCs neither from young nor old mice. Next, we evaluated the ultrastructure of caveolae in young versus old VSMCs. Although caveolae were present in cells of both groups, the density of caveolae was reduced in old VSMCs compared to young VSMCs (Figure 3g–i). We next confirmed our results by using a novel *EHD2* genetic knockout (KO) mouse model. Since EHD2 localizes to the caveolar neck region of all caveolae, genetic abolition of EHD2 increases ubiquitously detachment of caveolae from the plasma membrane (Matthaeus et al., 2019). In line with these findings, we found detachment of caveolae in *EHD2* del/del VSMCs compared to control VSMCs (Figure 4a). These changes were accompanied by reduced expression of Ca_v_3.2 channels in *EHD2* KO (del/del) VSMCs compared to control cells. Furthermore, Ca^2+^ spark frequency and the percentage of cells firing Ca^2+^ sparks were diminished in VSMCs from *EHD2* del/del mice (Figure 4). Together, ultrastructural alterations of caveolae, reduced expression of Ca_v_3.2 channels or both could underlie the observed attenuation of the vascular T‐type Ca_V_3.2‐RyR axis to generate Ca^2+^ sparks in aged vascular smooth muscle.

### 2.4 Residual Ca^2+^ sparks in aged VSMCs

**Figure 5:**
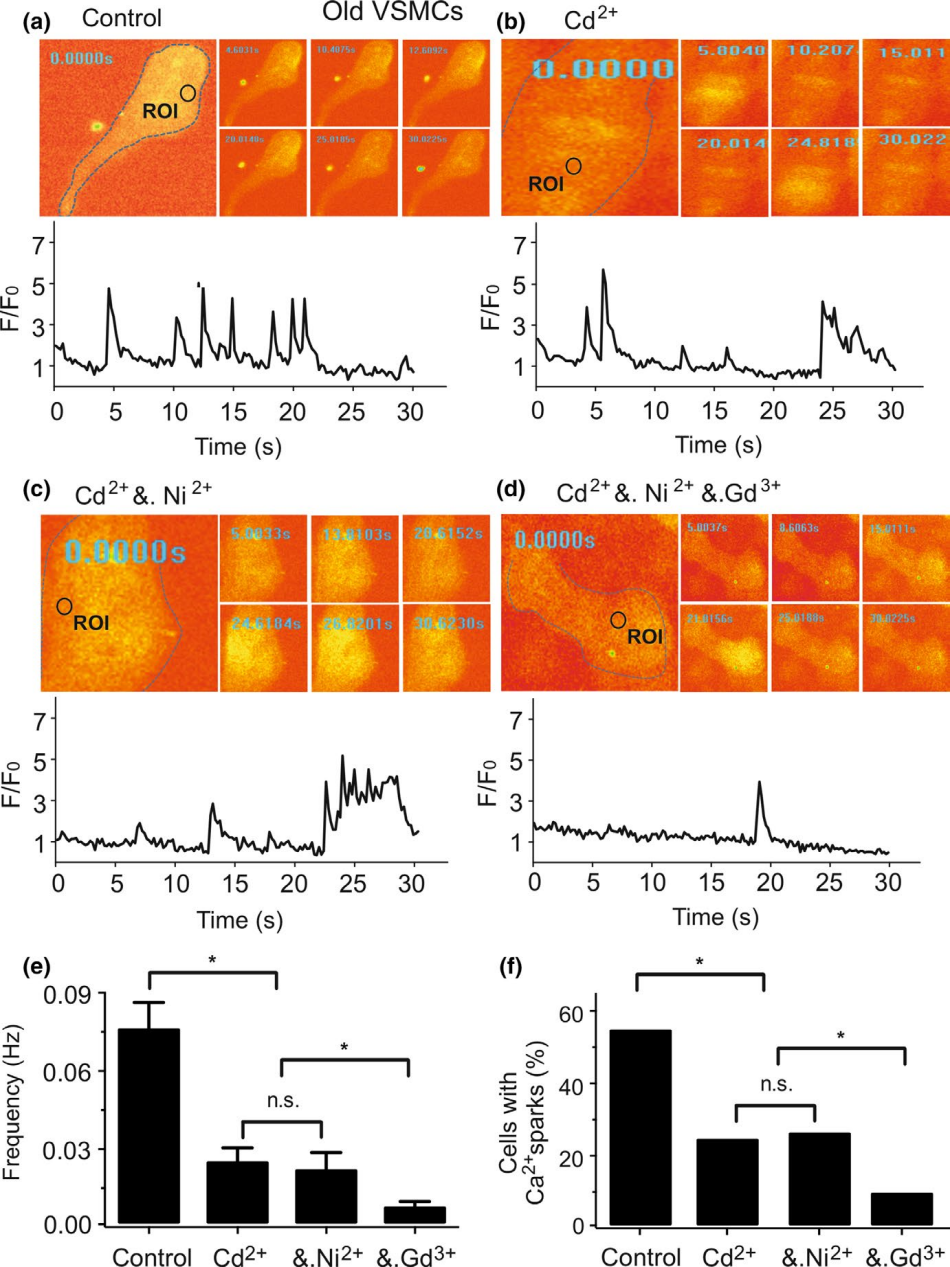
Gd^3+^ sensitive (TRP) cation channels generate Ca^2+^ sparks in old VSMCs. (a), Ca^2+^ fluorescence images of a Fluo‐4‐AM–loaded VSMC from an old mouse and time course of Ca^2+^ fluorescence changes in the cellular ROI (upper panel). Cell boundary is marked with dashed line. (b), same as (a) but with a cell incubated with Cd^2+^ (200 µM). (c), same as (a) but with Ni^2+^ (50 µM) following Cd^2+^ treatment. (d), same as (a) but with Gd^3+^ (100 µM) following Cd^2+^+ Ni^2+^ treatment. (e, f), summary of the results. Ca^2+^ spark frequency (e) and fraction of cells producing Ca^2+^ sparks (f) in cells (*n* = 66), in cells incubated with Cd^2+^ (*n* = 69), in cells incubated with Cd^2+^+ Ni^2+^ (*n* = 61), and in cells incubated with Cd^2+^+ Ni^2+^+ Gd^3+^ (*n* = 86). Cells were isolated from 4 old mice in each group; 15–30 cells were recorded and analyzed from each mouse. *, *p* < .05. n.s., not significant

We noticed that there was a fraction of Ca^2+^ sparks in old VSMCs, which was insensitive to Ca_V_1.2 and Ca_V_3.2 channel blockade by Cd^2+^ and Ni^2+^, respectively (Figure 5). Surprisingly, Gd^3+^, a permissive TRP channel blocker, inhibited these remaining Ca^2+^ sparks (Figure 5). In contrast, Gd^3+^ (100 µM) had no effects on Ca^2+^ sparks in young VSMCs (Figure S1l,m). Together, the data suggest that defective Ca_V_3.2‐RyR coupling in old VSMCs is counterbalanced by putative Gd^3+^ sensitive (TRP) cation channels providing sufficient Ca^2+^ influx to generate Ca^2+^ sparks.

### 2.5 Age‐dependent regulation of myogenic tone by Cav3.2 channels

**Figure 6:**
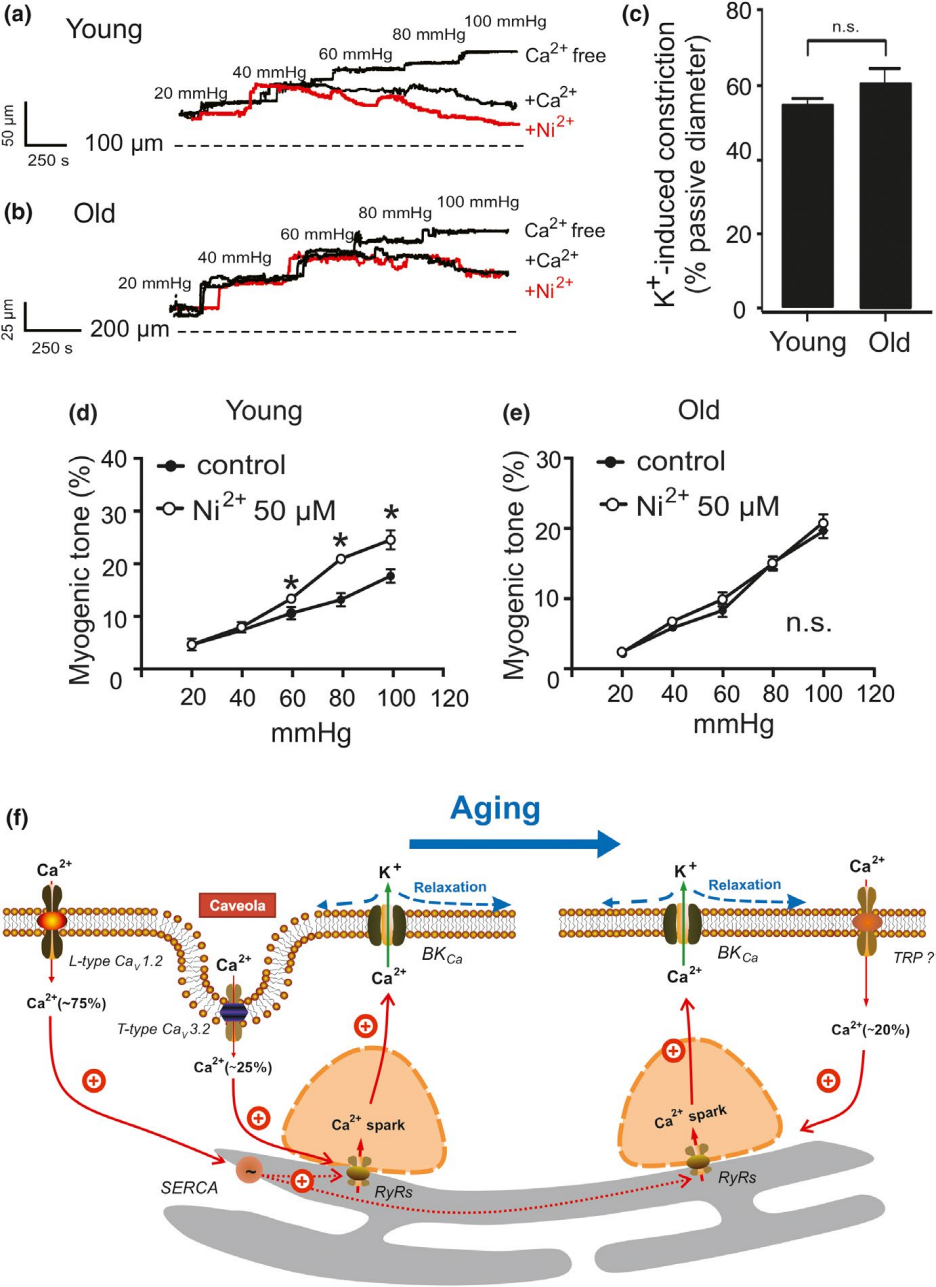
T‐type Ca_V_3.2 blockade does not constrict mesenteric arteries from old mice. (a, b), representative traces and summary data show the effect of Ni^2+^ (50 µM) on mesenteric arteries pressurized to 60–100 mmHg from young and old mice, respectively. (c), vasoconstriction evoked by 60 mM K^+^ was similar in young and old pressurized (15 mmHg) arteries. (d, e), summary of myogenic tone measurements in pressurized mesenteric arteries from young and old mice (*n* = 5 arteries from 5 mice, one artery was recorded and analyzed from each mouse). Experiments were performed in the absence and presence of 50 µM Ni^2+^. *, *p* < .05. n.s., not significant. (f), schematic illustration of major Ca^2+^ influx pathways regulating Ca^2+^ sparks in VSMCs during aging. Ca^2+^ sparks, which result from opening of clustered RyRs in the SR, activate large‐conductance Ca^2+^‐activated K^+^ (BK_Ca_) channels to produce a negative feedback effect on vasoconstriction. L‐type Ca_v_1.2 channels contribute to global cytosolic [Ca^2+^], which in turn influences luminal SR calcium (*via* SERCA) and thus generates the majority (75%) of Ca^2+^ sparks. Caveolae position Ca_V_3.2 channels sufficiently close to RyRs for extracellular Ca^2+^ influx to trigger (~25%) Ca^2+^ sparks. In aged mice, this Ca_V_3.2‐RyR pathway loses importance. Instead, a gadolinium‐sensitive Ca^2+^ influx pathway is upregulated to trigger (20%) Ca^2+^ sparks. This pathway may compromise nonselective TRP channels. RyRs, ryanodine receptors; SERCA, sarcoplasmic/endoplasmic calcium pump; SR, sarcoplasmic reticulum; VSMC, mesenteric artery vascular smooth muscle cell

To ascertain the importance of the Ca_V_3.2‐RyR relationship to regulate arterial tone, we performed video microscopic measurements on isolated arteries. In young wild‐type mesenteric arteries, the Ca_v_3.2 blocker Ni^2+^ 50 µM increased myogenic tone from 9.2% ± 1.2% to 13.04% ± 0.8% at 60 mmHg, from 11.6% ± 1.2% to 19.7% ± 0.5% at 80 mmHg, and from 17.7% ± 2% to 27.8% ± 1.3% at 100 mmHg (Figure 6), whereas Ni^2+^ 50 µM did not affect myogenic constriction in old vessels. Despite these differences, 60 mM K^+^‐induced vasoconstrictions were similar between young (54.2% ± 1.2%) and old (60.7% ± 2.1%) pressurized arteries (Figure 6c).

## 3 DISCUSSION

In this study, we analyzed the effects of aging on the Ca_v_3.2 channels‐RyR axis on Ca^2+^ sparks generation in VSMCs. We employed pharmacological tools, smooth muscle‐specific Ca_v_1.2 channel (SMAKO) and *EHD2* genetic knockout mice. Our studies demonstrate that caveolar Ca_v_3.2 channels‐RyR axis is impaired in aged VSMCs. We observed age‐related ultrastructural alterations of caveolae, which together with decreased Ca_v_3.2 expression, may underlie incomplete caveolae‐Ca_v_3.2‐RyR coupling for extracellular Ca^2+^ influx to trigger Ca^2+^ sparks and BK_Ca_ feedback in aged vascular smooth muscle.

### 3.1 Local and tight caveolar Ca_V_3.2‐RyR coupling

L‐type Ca_v_1.2 channels provide the predominant Ca^2+^ pathway for Ca^2+^ spark generation in VSMCs (Brenner et al., 2000; Filosa et al., 2006; Gollasch et al., 1998; Nelson et al., 1995; Pluger et al., 2000; Sausbier et al., 2005). This pathway increases Ca^2+^ load in the SR ([Ca^2+^]_SR_) can activate RyRs from the SR luminal side of the receptor to produce Ca^2+^ sparks (Figure 6f) (Ching, Williams, & Sitsapesan, 2000; Essin et al., 2007). T‐type Ca_v_3.2 channels, which are located in pits structures of caveolae, constitute an additional Ca^2+^ influx pathway to trigger Ca^2+^ sparks (Figure 6f) (Abd El‐Rahman et al., 2013; Braunstein et al., 2009; Chen et al., 2003; Fan et al., 2018; Hashad et al., 2018). Our recent data show that RyR2 is the predominant RyR isoform responsible for Ca^2+^ sparks in VSMCs (Kassmann et al., 2019). The results from the present study are in line with these conceptual views. We first used low concentrations of the SR Ca^2+^‐ATPase inhibitor thapsigargin to decrease the SR calcium content (Janczewski & Lakatta, 1993; Lewartowski & Wolska, 1993; Nelson et al., 1995; Sagara, Fernandez‐Belda, Meis, & Inesi, 1992) and found that [Ca^2+^]_SR_ depletion reduced Ca^2+^ spark frequency. In contrast, thapsigargin did not affect Ca^2+^ spark frequency in the absence of Ca_v_1.2 channels. These data indicate that SR calcium filling through SERCA is critical for Ca_V_1.2‐mediated Ca^2+^ sparks, but not for Ca_V_3.2‐RyR axis. They support that local and tight coupling between the Ca_V_1.2 channels and RyRs is not required to initiate Ca^2+^ sparks as previously suggested by our group (Essin et al., 2007). Indeed, the data indicate that Ca_v_1.2 channels contribute to global cytosolic [Ca^2+^], which in turn influences luminal SR calcium and thus Ca^2+^ sparks (Figure 6f) (Essin et al., 2007). We also found that Ca_v_3.2 channel blockade by Ni^2+^ had no effects on Ca^2+^ sparks and STOCs after treatment of cells with thapsigargin, that is, in conditions of suboptimal filled [Ca^2+^]_SR_ stores. These data indicate that proper function of the caveolar T‐type Ca_V_3.2‐RyR axis requires sufficient high [Ca^2+^]_SR_ load. Second, we also explored the function of Ca_v_1.2 and Ca_v_3.2 channels for luminal SR Ca^2+^ load. We used high concentrations of caffeine (10 mM), a well‐known activator RyRs, to induce SR calcium release. Caffeine evoked smaller Ca^2+^ transients through SR Ca^2+^ release in SMAKO Ca_v_1.2^−/−^ VSMCs, in which T‐type Ca_v_3.2 channels play a minor role in providing Ca^2+^ influx to induce Ca^2+^ sparks. These findings support the view that Ca^2+^ influx through L‐type Ca_v_1.2 channels, but not T‐type Ca_v_3.2 channels, represents the main source for luminal SR calcium load (Essin et al., 2007; Fan et al., 2018). To confirm this conclusion, we studied Ca^2+^ uptake into luminal SR by 2 pulse‐protocol of caffeine applications. We found that 10 mM caffeine evoked weak caffeine‐induced peaks in SMAKO Ca_v_1.2^−/−^ cells compared to control cells fifteen minutes after the 1st‐pulse caffeine application. We failed to observe Ca^2+^ sparks in SMAKO Ca_v_1.2^−/−^ cells before the 2nd‐pulse caffeine application, whereas cells with functional Ca_v_1.2 channels enabled generation of Ca^2+^ sparks within the fifteen minutes interval. The poor recovery of the luminal SR calcium in SMAKO Ca_v_1.2^−/−^ VSMCs suggests that T‐type Ca_v_3.2 channels play a minor role in [Ca^2+^]_SR_ filling. The results were also confirmed by our electrophysiological experiments.

### 3.2 Effects of aging on T‐type Ca_V_3.2‐RyR axis

In order to explore the effects of aging on caveolar T‐type Ca_v_3.2 channel‐mediated Ca^2+^ sparks, we treated VSMCs from young and old mice with Ni^2+^ and methyl‐ß‐cyclodextrin. Consistent with our previous findings (Fan et al., 2018; Hashad et al., 2018), both compounds inhibited Ca^2+^ sparks in young VSMCs. In contrast, neither Ni^2+^ nor methyl‐ß‐cyclodextrin inhibited Ca^2+^ sparks in old VSMCs. These results indicate that the T‐type Ca_V_3.2‐RyR axis loses its function to generate Ca^2+^ sparks in aged VSMCs to drive negative feedback control of myogenic tone in resistance arteries (Figure 6a‐e). The data are consistent with other data showing that Ca_V_3.2 channels lose their protective role against excess myogenic tone and the loss of Ca_V_3.2 channels induces a loss of flow‐mediated vasodilation with advanced age (Mikkelsen et al., 2016). Since RyR2 is the predominant RyR isoform responsible for Ca^2+^ sparks in VSMCs (Kassmann et al., 2019) and Ca_V_1.2‐RyR2 axis works efficient in old VSMCs (Figure 5), RyRs reorganization should not be a key reason for altered calcium sparks in aged VSMCs. Thus, we propose that the observed malfunction of T‐type Ca_V_3.2‐RyR axis in aging results from reduced Ca_V_3.2 expression and ultrastructural alterations in caveolar microdomains responsible for Ca_V_3.2‐RyR coupling. In accordance, we found that caveolae density was decreased and caveolae necks were narrowed in old VSMCs. T‐type Ca_V_3.2‐RyR axis provides an important vascular Ca^2+^ influx pathway for triggering Ca^2+^ sparks in young VSMCs that deserves further attention since Ca_V_3.2 T‐type calcium channels contribute to cardiovascular diseases (Chiang et al., 2009; David et al., 2010). Defective T‐type Ca_V_3.2‐RyR axis may contribute to age‐related cardiovascular complications involving increased myogenic tone and blood pressure with advanced age.

### 3.3 Role of EHD2 on T‐type Ca_V_3.2‐RyR axis

EHD2 is a dynamin‐related ATPase located at the neck of caveolae, which constitutes a structural component of caveolae involved in controlling the stability and turnover of this organelle (Ludwig et al., 2013; Morén et al., 2012; Stoeber et al., 2016). Knockout or down‐regulation of *EHD2* in vivo results in decreased surface association and increased mobility of caveolae, whereas EHD2 overexpression stabilizes caveolae at the plasma membrane (Matthaeus et al., 2019; Morén et al., 2012; Shvets, Bitsikas, Howard, Hansen, & Nichols, 2015; Stoeber et al., 2016). Here we used *EHD* del/del mice to disturb the stability of caveolae to explore the effect of caveolar microdomains on Ca_V_3.2‐RyR axis. Loss of EHD2 decreased the plasma membrane localization of caveolae and Ca_V_3.2 channel expression, thus impaired the ability of T‐type Ca_V_3.2 on Ca^2+^ sparks generation in the mesenteric SMC. It aligns with our above results and provides firm evidence that Ca_V_3.2 channels in caveolar microdomains contribute to Ca^2+^ sparks in VSMCs of young but not old mice.

### 3.4 Possible role of TRP channels

We found that complete blockade of both Ca_V_1.2 and Ca_V_3.2 channels (by Cd^2+^ and Ni^2+^) abolished all Ca^2+^ sparks in young VSCMs (see also Fan et al., 2018) but only ~70% of Ca^2+^ sparks in old VSMCs. The findings suggest appearance of an additional Ca^2+^ influx pathway evoking Ca^2+^ sparks only in aged VSMCs. We found that gadolinium, a permissive TRP channel blocker (Hashad et al., 2017; Riehle et al., 2016), inhibited these remaining Ca^2+^ sparks. In order to rule out possible effects of gadolinium on Ca_v_1.2 channel and/or Ca_v_3.2 channel‐mediated Ca^2+^ sparks, we tested the effects of gadolinium on Ca^2+^ sparks in young VSMCs (in the absence of Cd^2+^ and Ni^2+^) and found that gadolinium had no effects on these Ca^2+^ sparks. Although gadolinium has been identified as nonspecific blocker (Berrier, Coulombe, Szabo, Zoratti, & Ghazi, 1992; Gottlieb, Suchyna, Ostrow, & Sachs, 2004; Trollinger, Rivkah Isseroff, & Nuccitelli, 2002), it is likely that a Ca^2+^ permeable conductance (TRP channels) has been upregulated to compensate for loss of T‐type Ca_V_3.2 channels driving Ca^2+^ sparks in aged VSMCs (Figure 6f). Besides, TRP channels might trigger calcium sparks through reloading the SR with calcium since methyl‐ß‐cyclodextrin treatment failed to alter calcium events in old VSMCs (Figure 3e,f). Further works are required to ascertain which TRP cation channel(s) or pathways are responsible for generation of these Ca^2+^ sparks. Identification of the underlying pathways might be important for understanding age‐dependent factors contributing to cardiovascular disease and providing novel therapeutic approaches.

### 3.5 Summary

Our data provide further evidence that Ca_V_3.2 channels colocalize in microdomains with RyRs to initiate Ca^2+^ sparks and activate BKCa channels to drive a feedback response on vascular tone. Here we demonstrate that caveolar Ca_V_3.2 channels are impaired in triggering Ca^2+^ sparks in aged VSMCs. This defective caveolae‐RyR coupling may be caused by age‐related ultrastructural alterations of caveolae and reduced Ca_V_3.2 expression in VSMCs. Furthermore, we found that proper function of the T‐type Ca_V_3.2‐RyR axis requires sufficiently high SR Ca^2+^ load, which is regulated *via* Ca^2+^ influx through L‐type Ca_V_1.2 channels. T‐type Ca_V_3.2‐RyR axis malfunction may provide a straightforward explanation on how aging affects blood pressure (Chiossi et al., 2016; Hilgers et al., 2017; Wirth et al., 2016). Targeting defective Ca_V_3.2‐RyR coupling may provide new therapeutic avenues for treatment of cardiovascular disease in the elderly.

## 4 EXPERIMENTAL PROCEDURES

### 4.1 Mice

In this study, young (12–14 weeks) versus old (48–56 weeks) male mice were used. The generation and usage of mice deficient in the smooth muscle Ca_v_1.2 Ca^2+^ channel (SMAKO, smooth muscle α1c‐subunit Ca^2+^ channel knockout) has been described previously (Moosmang et al., 2003). Briefly, a conditional lox P‐flanked allele (L2) of the Ca_v_1.2 gene (i.e., exons 14 and 15) was generated by homologous recombination in R1 embryonic stem cells (Seisenberger et al., 2000). In addition, mice carried a knock‐in allele (SM‐CreER T2 (ki)) (Kuhbandner et al., 2000), which expresses the tamoxifen‐dependent Cre recombinase, CreER T2, from the endogenous SM22 α gene locus, which is selectively expressed in smooth muscle of adult mice. Thus, tamoxifen treatment results in conversion of the lox P‐flanked Ca_v_1.2 allele (L2) into a Ca_v_1.2 knockout allele (L1) specifically in SMCs (Moosmang et al., 2003) (Essin et al., 2007). Mice were maintained at the breeding facility of the Max Delbrück Center for Molecular Medicine Berlin (MDC) in individually ventilated cages under standardized conditions that included a 12‐hr dark‐light cycle and free access to standard chow (0.25% sodium; SSNIFF Spezialitäten, Soest, Germany) and drinking water. SMAKO mice (*Ca_v_1.2* ^flox/flox^; SM22α‐Cre^T2^ or *Ca_v_1.2* ^flox/flox^; SM22α‐Cre^T2/T2^) and corresponding control mice (*Ca_v_1.2* ^+/+^; SM22α‐Cre^T2/T2^, *Ca_v_1.2* ^+/+^; SM22α‐Cre^T2^, *Ca_v_1.2* ^+/+^ or *Ca_v_1.2* ^flox/flox^) (12–14 weeks each) were i.p. injected with tamoxifen (30 µg/g body weight/day) for five consecutive days and sacrificed within 2–4 days after the injections. *EHD2* del/del or *EHD2* del/+ littermates (as control) mice (20–35 weeks each) were used as previously described (Matthaeus et al., 2019). All mice were deeply anesthetized by inhalation of isoflurane until cessation of breathing and then killed by cervical dislocation, and the mesentery arteries were removed. Experiments were performed on the same day with arteries from litter‐matched young versus aged mice, *EHD2* control versus *EHD2* del/del, and control versus SMAKO mice. All animal protocols were approved by the local animal care committee (LAGeSo, Berlin, Germany) and the animal welfare officers of the MDC (No. X9011/16, G0154/14). There are no ethical concerns.

### 4.2 Isolation of arterial vascular smooth muscle cells

Arterial VSMCs from mesenteric arteries were isolated as previously described (Gollasch et al., 1998; Kassmann et al., 2019; Pluger et al., 2000; Schleifenbaum et al., 2014). Briefly, arteries were removed and quickly transferred to cold (4°C) oxygenated (95% O_2_‐5% CO_2_) physiological salt solution (PSS) of the following composition (mM): 119 NaCl, 4.7 KCl, 1.2 KH_2_PO_4_, 25 NaHCO_3_, 1.2 MgSO_4_, 1.6 CaCl_2_, and 11.1 glucose. The arteries were cleaned, cut into pieces, and placed into a Ca^2+^‐free Hank's solution (mM): 55 NaCl, 80 sodium glutamate, 5.6 KCl, 2 MgCl_2_, 1 mg/ml bovine serum albumin (BSA, Sigma, Taufkirchen), 10 glucose, and 10 HEPES (pH 7.4 with NaOH) containing 0.5 mg/ml papain (Sigma) and 1.0 mg/ml DTT for 37 min at 37°C. The segments were then placed in Hank's solution containing 1 mg/ml collagenase (Sigma, type F and H, ratio 30% and 70%) and 0.1 mM CaCl_2_ for 17 min at 37°C. Following several washes in Ca^2+^‐free Hank's solution (containing 1 mg/ml BSA), single cells were dispersed from artery segments by gentle triturating. Cells were then stored in the same solution at 4°C.

### 4.3 Ca^2+^ imaging measurements

Ca^2+^ sparks were measured as previously described (Essin et al., 2007; Fan et al., 2018). Isolated VSMCs were placed onto glass coverslips and incubated with the Ca^2+^ indicator fluo‐4 a.m. (10 µM) and pluronic acid (0.005%, w/v) for 60 min at room temperature in Ca^2+^‐free Hanks’ solution (Fan et al., 2018; Kassmann et al., 2019). After loading, cells were washed with bath solution for 10 min at room temperature. Isolated cells and intact arterial segments were imaged in a bath solution containing (mM): 134 NaCl, 6 KCl, 1 MgCl_2_, 2 CaCl_2_, 10 glucose and 10 HEPES (pH 7.4, NaOH). Images were recorded using a Nipkow disc‐based UltraView LCI confocal scanner (Perkin Elmer, Waltham, MA, USA) linked to a fast digital camera (Hamamatsu Photonics Model C4742‐95‐12ERG, 1,344 × 1,024 active pixel resolution, 6.45 µm square pixels). The confocal system was mounted on an inverted Nikon Eclipse Ti microscope with a x40 oil‐immersion objective (NA 1.3, Nikon). Images were obtained by illumination with an argon laser at 488 nm and recording all emitted light above 515 nm. Ca^2+^ spark analyses were performed off‐line using the UltraView Imaging Suite software (Perkin Elmer). The entire area of each image was analyzed to detect Ca^2+^ sparks. Ca^2+^ sparks were defined as local fractional fluorescence increase (*F*/*F*_0_) above the noise level of 1.5. The frequency was calculated as the number of detected sparks divided by the total scan time. Caffeine‐induced peak was measured as previously described (Fernandez‐Sanz et al., 2014). After the VSMCs loaded with Ca^2+^ indicator fluo‐4 a.m. (10 µM, 60 min at room temperature), images were obtained following a single pulse of 10 mM caffeine. Maximal amplitude of caffeine‐induced peak fluorescence was normalized by the initial fluorescence value (*F/F_0_*) and considered as an index of total SR Ca^2+^ load.

### 4.4 Electrophysiology

Currents were measured in the whole‐cell perforated‐patch mode of the patch‐clamp technique (Essin et al., 2007; Gollasch, Ried, Bychkov, Luft, & Haller, 1996; Kassmann et al., 2019). Patch pipettes (resistance, 1.5–3.5 MΩ) were filled with a solution containing (in mM): 110 K‐Asp, 30 KCl, 10 NaCl, 1 MgCl_2_, and 0.05 EGTA (pH 7.2). The patch pipette solution was supplemented with 200 µg/ml Amphotericin B, dissolved in dimethyl sulfoxide (DMSO), to measure K^+^ currents in the whole‐cell perforated‐patch mode. The external bath solution contained (in mM): 134 NaCl, 6 KCl, 1 MgCl_2_, 2 CaCl_2_, 10 glucose, and 10 HEPES (pH 7.4); holding potential was −60 mV. Whole‐cell currents were recorded using an Axopatch 200B amplifier (Axon Instruments/Molecular Devices) or an EPC 7 amplifier (List) at room temperature. Data were digitized at 5 kHz, using a Digidata 1440A digitizer (Axon CNS, Molecular Devices) and pClamp software versions 10.1 and 10.2. STOC analysis was performed off‐line using IGOR Pro (WaveMetrics) and Microsoft Excel software (Microsoft Corporation). A STOC was identified as a signal with at least three times the BK_Ca_ single‐channel current amplitude (Kassmann et al., 2019).

### 4.5 Ultrastructure and quantitative assessment of caveolae

Quantitative assessment of caveolae was carried out as previously described (Lowalekar et al., 2012). Isolated VSMCs from mesenteric arteries were dehydrated in a graded series of ethanol and embedded in the PolyBed® 812 resin (Polysciences Europe GmbH), ultrathin sections (60–80 nm) were cut (Leica microsystems), and uranyl acetate and lead citrate staining was performed. Samples were examined at 80 kV with a Zeiss EM 910 electron microscope (Zeiss), and image acquisition was performed with a Quemesa CDD camera and the iTEM software (Emsis GmbH). The density of caveolae was calculated as number of caveolae per µm. The diameter of caveolae neck (nm) and caveolae size (nm) were determined by using the parallel dimension function of CorelDRAW. Values from all electron microscopy images (*n* = 18 cells in each group) were averaged for each group.

### 4.6 Western blot analysis

Mesenteric arteries were isolated from mice and placed into cold physiological saline solution (PSS) previously oxygenated for 30 min (95% O_2_, 5% CO_2_). Vessels were cleaned of perivascular fat, and all tissues were immediately placed on dry ice and kept at −80°C until use. Samples were homogenized in RIPA buffer (Cell Signaling Technology) containing protease inhibitors (Sigma‐Aldrich). Tubes containing homogenates were freeze‐thawed three times at −80°C and 37°C, respectively, and then centrifuged at 11,200 *g* for 20 min at 4°C. After determining protein concentration, samples prepared in Laemmli buffer (50 mM Tris pH 6.8, 10% SDS, 10% glycerol, 5% mercaptoethanol, and 2 mg/ml bromophenol blue) were boiled for 2 min, separated by sodium dodecyl sulfate–polyacrylamide gel electrophoresis (SDS‐PAGE) on 7% polyacrylamide gels and transferred onto polyvinylidene fluoride membranes. Membranes were blocked in 5% nonfat dry milk in phosphate‐buffered saline (PBS) containing 0.1% Tween 20 and then incubated overnight at 4°C with primary anti‐Ca_V_3.2 antibody (Mouse. NBP1‐22444, 1:1,000 final dilution; Novus Biologicals). After washing, membranes were incubated with anti‐mouse IgG‐peroxidase‐linked secondary antibody (1:5,000 final dilution; GE Healthcare) for 1 hr at room temperature. Blots were washed and incubated in enhanced chemiluminescence reagents (ECL Prime, Amersham Bioscience), after which bands were detected using a ChemiDoc XRS+ Imaging System (Bio‐Rad). An anti‐Actin antibody (Mouse. sc‐8432, 1:500 final dilution; Santa Cruz) was used as a loading control, and Precision Plus Protein Prestained Standard (Bio‐Rad) was used as a molecular weight marker.

### 4.7 Immunohistostaining of mesenteric arteries for confocal imaging

*EHD2* del/+ and *EHD2* del/del mice were anesthetized with 2% ketamine/10% rompun, perfused by 30 ml PBS and 50 ml 4% PFA (Roth, diluted in PBS), and afterward, vessels were dissected, and tissue pieces were further fixed for 4 hr in 4% PFA, transferred to 15% sucrose (in PBS, Merck) for 4 hr and incubated in 30% sucrose overnight. After embedding in TissueTek (Sakura), the tissue is frozen at −80°C and 8‐µm sections were obtained in a Leica cryostat at −30°C. For immunostainings, the cryostat sections were incubated with blocking buffer (1% donkey serum/1% Triton X‐100/PBS), the first antibody was applied overnight at 4°C, and after washing with PBS/1% Tween, the secondary antibody and DAPI were applied for 2 hr. Afterward, the sections were embedded in ImmoMount (Thermo Scientific #9990402). The stained sections were analyzed with Zeiss LSM700 microscope provided with Zeiss 40x objective, and images were analyzed by ImageJ/Fij. Antibodies: anti‐beta‐actin‐mouse (Sigma #A2228), anti‐Cav3.2‐rabbit (Alomone Labs #ACC‐025), anti‐mouse‐Alexa488 (Invitrogen #R37114), anti‐rabbit‐Cy3 (Dianova #711‐165–152), and DAPI (Sigma #D9542).

### 4.8 Vessel myography

Vessel myography was performed as previously described (Schleifenbaum et al., 2014) (Kassmann et al., 2019). Mesenteric arteries (third or fourth order) were mounted on glass cannula and superfused continuously with physiological saline solution (95% O_2_ −5% CO_2_; pH, 7.4; 37°C) containing (mM): 119 NaCl, 4.7 KCl, 25 NaHCO_3_, 1.2 KH_2_PO_4_, 1.6 CaCl_2_, 1.2 MgSO_4_, and 11.1 glucose. The intravascular pressure was incrementally elevated from 20 to 100 mmHg using a pressure servo control system (Living System Instrumentation), and the inner diameter of the vessel was measured (Nikon Diaphot). The recording system was connected to a personal computer for data acquisition and analysis (HaSoTec). Arteries were equilibrated at 15 mmHg for 60 min and contractile responsiveness assessed by applying 60 mM KCl before starting experiments.

### 4.9 Materials

Fluo‐4‐AM was purchased from Molecular Probes (Eugene). Thapsigargin was purchased from Alomone Laboratories. All salts and other drugs were obtained from Sigma‐Aldrich or Merck. In cases where DMSO was used as a solvent, the maximal DMSO concentration after application did not exceed 0.5% (Kassmann et al., 2019; Tsvetkov et al., 2016).

### 4.10 Statistics

Data are presented as means ± *SEM*. Statistically significant differences in mean values were determined by Student's unpaired *t* test or one‐way analysis of variance (ANOVA) or Mann–Whitney *U* test. *p*‐values < .05 were considered statistically significant; “*n*” represents the number of cells.

## CONFLICT OF INTEREST

None declared.

## AUTHOR CONTRIBUTIONS

G.F., M.K., Y.C., D.T, C.M., S.K., C.Z., S.Z., and Y.X. were responsible for data collection, analysis, and interpretation. M.K. and M.G. were responsible for the conception and design of the experiments. G.F. and M.G. drafted the manuscript. All authors were responsible for interpretation of the data, contributed to the drafting, and revised the manuscript critically for important intellectual content. All authors have approved the final version of the manuscript and agreed to be accountable for all aspects of the work. All persons designated as authors qualify for authorship, and all those who qualify for authorship are listed.

## Supporting information

Fig S1

## Data Availability

I confirm that my article contains a Data Availability Statement even if no new data was generated (list of sample statements) unless my article type does not require one.
