# Supplementary material for: Age attenuates the T‐type CaV3.2‐RyR axis in vascular smooth muscle
Source: Aging Cell. 2020 Mar 18;19(4):e13134. doi: 10.1111/acel.13134 (PMC7189999; doi:10.1111/acel.13134)
Supplement: Supplementary file 1 — Fig S1 [file ACEL-19-e13134-s001.docx]

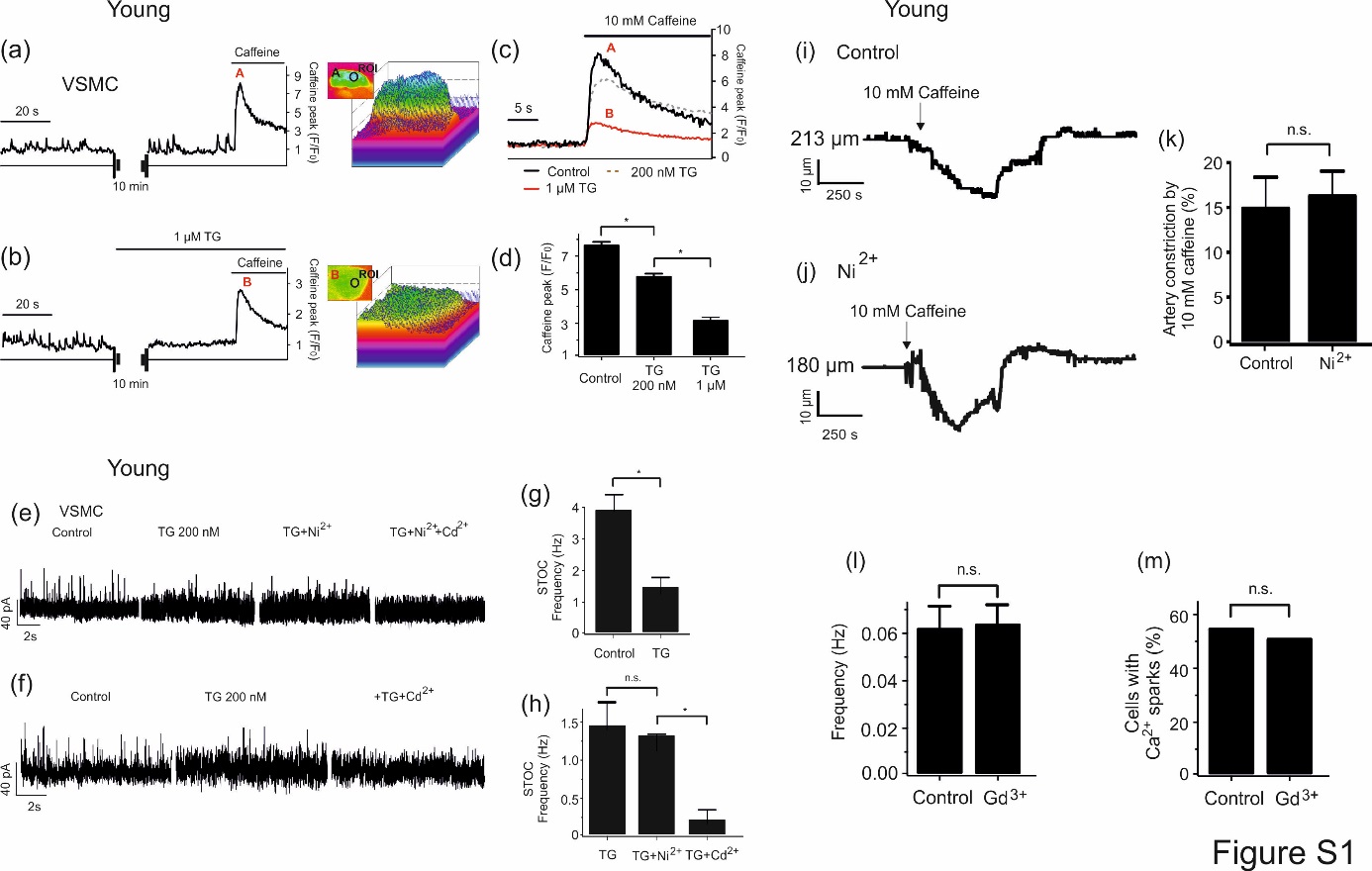


**Supplementary Figure S1a-d. Effects of thapsigargin on caffeine-induced Ca^2+^ release.** **(a),** Time course of caffeine-induced (10 mM) Ca^2+^ fluorescence changes (left panel) in the cellular ROI of the Ca^2+^ fluorescence image (right panel) from a Fluo-4-AM–loaded wild-type VSMC. **(b)**, same as (a) but in the presence of thapsigargin (1 µM). **(c) and (d),** effect of different concentrations of thapsigargin on caffeine peaks in wild-type VSMCs. **(c),** time course of Ca^2+^ fluorescence changes of caffeine peaks in the cellular ROI under different concentrations of thapsigargin (200 nM and 1 µM). **(d),** summary of the caffeine-induced Ca^2+^ peaks observed in the presence of different concentrations of thapsigargin (200 nM and 1 µM). n=7 cells from 3 mice, 2-3 cells were recorded and analyzed from each mouse. Thapsigargin, TG. *, *P*<0.05.

**Supplementary Figure S1e-h. Effects of Cd^2+^ and Ni^2+^ on STOCs in the presence of thapsigargin. (e),** Original recordings of STOCs in VSMCs before and after application of thapsigargin (200 nM), +Ni^2+^ (50 µM), and/or +Cd^2+^ (200 µM). Holding potential was -40 mV. **(f)**, same as **(e)**, but the cells treated with thapsigargin (200 nM), and +Cd^2+^ (200 µM). **(g)- (h)**, summary of the results. STOC frequencies (**g**), and amplitudes of STOCs (**h**) in each group cells (n=4 cells from 4 mice, one cell was recorded and analyzed from each mouse). *, *P*<0.05. n.s., not significant.

**Supplementary Figure S1i-k. Effects of Ni^2+^ on caffeine induced constrictions. (i, j),** representative traces and summary data show the effects of caffeine (10 mM) on mesenteric arteries pressurized to 80 mmHg. The arteries were isolated from young mice. Experiments were performed in the absence and presence of 50 µM Ni^2+^. **(k)**, summary of myogenic tone measurements in pressurized mesenteric arteries treated with or without Ni^2+^ (n=4 arteries from 4 mice, one artery was recorded and analyzed from each mouse). *, *P*<0.05. n.s., not significant.

**Supplementary Figure S1l, m.** Effects of Gd^3+^ (100 µM) on Ca^2+^ sparks in young VSMCs. Summary of the results. Ca^2+^ spark frequency (**i**) and fraction of cells producing Ca^2+^ sparks (**j**) in VSMCs from young mice in the absence (n=77) and presence of Gd^3+^ (100 µM) (n=87). Cells were isolated from 3 mice in each group; 20-35 cells were recorded and analyzed from each mouse. VSMC, vascular smooth muscle cell. n.s., not significant.
